# Differential Diagnosis of Frontotemporal Dementia, Alzheimer's Disease, and Normal Aging Using a Multi-Scale Multi-Type Feature Generative Adversarial Deep Neural Network on Structural Magnetic Resonance Images

**DOI:** 10.3389/fnins.2020.00853

**Published:** 2020-10-22

**Authors:** Da Ma, Donghuan Lu, Karteek Popuri, Lei Wang, Mirza Faisal Beg, Alzheimer's Disease Neuroimaging Initiative

**Affiliations:** ^1^School of Engineering Science, Simon Fraser University, Burnaby, BC, Canada; ^2^Tencent Jarvis X-Lab, Shenzhen, China; ^3^Feinberg School of Medicine, Northwestern University, Chicago, IL, United States

**Keywords:** differential diagnosis, magnetic resonance imaging, generative adversarial network, frontotemporal dementia (FTD), Alzheimer's disease

## Abstract

**Methods:** Alzheimer's disease and Frontotemporal dementia are the first and third most common forms of dementia. Due to their similar clinical symptoms, they are easily misdiagnosed as each other even with sophisticated clinical guidelines. For disease-specific intervention and treatment, it is essential to develop a computer-aided system to improve the accuracy of their differential diagnosis. Recent advances in deep learning have delivered some of the best performance for medical image recognition tasks. However, its application to the differential diagnosis of AD and FTD pathology has not been explored.

**Approach:** In this study, we proposed a novel deep learning based framework to distinguish between brain images of normal aging individuals and subjects with AD and FTD. Specifically, we combined the multi-scale and multi-type MRI-base image features with Generative Adversarial Network data augmentation technique to improve the differential diagnosis accuracy.

**Results:** Each of the multi-scale, multitype, and data augmentation methods improved the ability for differential diagnosis for both AD and FTD. A 10-fold cross validation experiment performed on a large sample of 1,954 images using the proposed framework achieved a high overall accuracy of 88.28%.

**Conclusions:** The salient contributions of this study are three-fold: (1) our experiments demonstrate that the combination of multiple structural features extracted at different scales with our proposed deep neural network yields superior performance than individual features; (2) we show that the use of Generative Adversarial Network for data augmentation could further improve the discriminant ability of the network regarding challenging tasks such as differentiating dementia sub-types; (3) and finally, we show that ensemble classifier strategy could make the network more robust and stable.

## 1. Introduction

As the first and third most common forms of dementia, Alzheimer's disease (AD) (Association et al., 2011) and Frontotemporal dementia (FTD) (Bang et al., 2015) are often mistaken as each other. This is due to the similarities in their clinical presentation, cognitive domains impairment, brain atrophy, and progressive alterations in language ability, behavior, and personality (Neary et al., 2005; Alladi et al., 2007; Womack et al., 2011). Despite significant efforts spent on establishing sophisticated clinical guidelines for their differential diagnosis, the diagnostic accuracy is still not satisfactory. Specifically, when diagnosing with the NINCDS-ADRDA criteria (Neary et al., 1998), the sensitivity of distinguishing AD subjects from FTD patients could reach as high as 93%; however, the specificity for FTD recognition is only 23% as most patients with FTD also fulfilled the NINCDS-ADRDA criteria for AD (Varma et al., 1999). With the necessity of applying different symptomatic intervention of treatment for various dementia subtypes in clinical practice (Pasquier, 2005), it is essential to develop a computer-aided diagnosis system for the improvement of the accuracy of differential diagnosis between these two dementias.

Patterns of brain atrophy observed in T1-weighted Magnetic Resonance Imaging (MRI) have been successfully used to capture structural changes in the human brain (Du et al., 2007; Davatzikos et al., 2011), specifically for using in developing computational systems that can identify the type of dementia pathology in the brain. Computer-aided diagnosis systems with MRI have been built for both AD and FTD (Suk et al., 2014; Jiskoot et al., 2018). In addition to binary classification with normal aging, T1-weighted MRIs have also been used for the differential diagnosis of AD and FTD by differentiating the atrophy pattern of these two types of dementia such as the affected regions and rate of change (Raamana et al., 2014). Various structural biomarkers have been explored to distinguish between AD and FTD, such as gray matter (GM) volume loss (Rabinovici et al., 2008), cortical thinning (Du et al., 2007), high-dimensional features based on GM and white matter (WM) volume distribution of whole brain (Davatzikos et al., 2008), as well as atrophy and shape deformity of individual structures (Looi et al., 2010).

Most previous studies on computer-aided diagnosis system for dementia classification emphasized on binary classification tasks, e.g., NC vs. FTD, NC vs. AD, or FTD vs. AD with few direct multi-class dementia classification methods in the literature. Raamana et al. compared multiple structural features, such as volumes, Laplacian invariants, and surface displacements of the hippocampus and lateral ventricle, regarding the multi-class classification among NC, AD, and FTD subjects (Raamana et al., 2014). With PCA and multi-class support vector machine (SVM) classifier, they achieved a 0.79 AUC. Tong et al. applied the RUSBoost algorithm (Seiffert et al., 2010) for the multi-class classification of subjective memory complaints, AD, frontotemporal lobe degeneration (FTLD), dementia with Lewy bodies, and vascular dementia (Tong et al., 2017). With volume and grading features as well as CSF measures and age, they achieved 75.2% overall accuracy with 0.8 sensitivity for AD and 0.63 sensitivity for FTLD.

Recently, deep learning has been delivering astounding performance for many recognition tasks (Hinton and Salakhutdinov, 2006; Krizhevsky et al., 2012; Simonyan and Zisserman, 2014). Its applications in computer-aided diagnosis has also drawn attention and it has out-performed traditional classification methods for many clinical recognition tasks (Suk et al., 2014; Ronneberger et al., 2015; Litjens et al., 2017). However, to the best of our knowledge, there have been no deep-learning-based approaches developed and published yet for the differential diagnosis of AD and FTD.

In this study, we proposed a novel framework to combine multi-type and multi-scale image-based features from structural MRI scans. Local volume size and surface thickness features were extracted by segmenting the T1-weighted MRI images into patches of a hierarchical size based on brain anatomy in a coarse-to-fine manner. A multi-scale and multi-type feature deep neural network (MMDNN) was developed to learn the latent representation across each individual features, along with the Generative Adversarial Network (GAN) technique for data augmentation and ensemble classifier strategy to increase robustness of the framework. A comprehensive validation experiment with 1,954 images demonstrates the superior performance of the proposed framework with 88.28% accuracy.

## 2. Methods

In the proposed framework, the original raw structural MRI images were first segmented into different anatomical structure region of interests (ROI) with FreeSurfer. Each ROI was further sub-clustered into smaller patches of super-pixels with multi-scales. The volume, cortical thickness at each level of the patch were extracted as multi-scale multi-type features. Finally, a Generative Adversarial Network with multi-type and multi-scale features was trained to achieve differential diagnosis to identify patients with AD and FTD from NC subjects.

### 2.1. Materials

Data used in this study were obtained from two publicly available databases, i.e., the Alzheimer's Disease Neuroimaging Initiative (ADNI) database adni.loni.usc.edu and the frontotemporal lobar degeneration neuroimaging initiative (NIFD) database http://memory.ucsf.edu/research/studies/nifd. The primary goal of ADNI is to test whether serial MRI, PET, other biological markers, and clinical and neuropsychological assessment can be combined to measure the progression of mild cognitive impairment (MCI) and early Alzheimer's disease (AD). Frontotemporal lobar degeneration Neuroimaging Initiative (FTLDNI), also referred to as NIFD started in 2010 with the primary goals being to identify neuroimaging modalities and methods of analysis for tracking frontotemporal lobar degeneration (FTLD) and to assess the value of imaging vs. other biomarkers in diagnostic roles. More detailed information about FTLDNI can be found in 4rtni-ftldni.ini.usc.edu.

Both ADNI and FTLDNI databases contain longitudinal scans for each participant. Subjects with who the diagnosis changes in any of their follow-up visits during the study period (i.e., MCI progressing to AD or reverting to NC), were excluded from the study to reduce the effect of potential misdiagnosis. A total of 1,954 Structural MRI were included in this study, 1,114 of which were from ADNI database, and the remaining 840 from the NIFD database. Table 1 shows the demographic and clinical information of these subjects in both database. The numbers in the brackets of the second row are the numbers of male and female subjects, while number before each bracket is the total number of subjects belong to that group. The numbers in the remaining three rows represent the mean and standard deviation of age, education, and MMSE, respectively.

**Table 1:** Demographic information of the subject included from the databases.

**Mean ± SD**	**NC**	**AD**	**FTD**
Count (M/F)	1063 (533/530)	459 (270/189)	434 (266/168)
Age (Mean ± SD)	72.19 ± 8.28	75.91 ± 7.54	64.69 ± 8.51
Education (Mean ± SD)	16.66 ± 3.24	15.13 ± 2.58	19.05 ± 1.12
MMSE (Mean ± SD)	29.40 ± 1.39	23.20 ± 1.96	25.36 ± 6.12

*First row (Count M/F): total number as well as number of male and female subjects in each clinically diagnostic group. Second row: the mean and standard deviation of subject age (unit: years). Third row: the mean and standard deviation of subject's years of education (unit: years). Forth row: the mean and standard deviation of subject's clinically evaluated MMSE score*.

### 2.2. Multi-Level Multi-Type Feature Extraction

For image recognition problems, convolutional neural network (CNN) and its variants, such as VGG16 (Simonyan and Zisserman, 2014), ResNet (He et al., 2016), and Inception-ResNet (Szegedy et al., 2017), have achieved the state-of-the-art performance in various tasks. However, those networks require a large number of labeled samples for their training. Especially with high dimensional data, as used in this study (256 × 256 × 256 3D images), larger kernel sizes or more layers are necessary to learn the latent representation, resulting in a larger network that needs even more training samples. The dataset used in our data set is considerably larger in magnitude than many other studies in the neuroimaging context, it is however still relatively small in scale as compared with most of the natural image recognition tasks. Therefore, to reduce the dimension of input data and the size of network, each MRI scan was segmented into small regions based on brain anatomy, which we denoted as “patches” hereafter, and two types of primary structural features, volume size and cortical mantle thickness, were extracted for the differential diagnosis of NC, AD, and FTD. For MRI scan segmentation and volume size feature extraction, the following steps were applied: (1) structural ROI parcellation, (2) Structural-wise patch cluster-based segmentation, (3) Feature extraction and normalization.

Firstly, in the ROI segmentation step, the gray matter (both cortical and subcortical) of each T1 structural MRI image was segmented into 87 anatomical ROIs using FreeSurfer 5.3 (Dale AM, 1999). For some ROIs, in particularly larger ones such as the occipital cortex, the discriminant information for brain structural change could be localized within the ROI to smaller focal locations. Such localized differences could potentially provide important information to differentiate AD and FTD but could be lost in aggregating the features across the whole ROI. Therefore, each ROI was further subdivided into smaller patches in the second patch parcellation step. Parcellation or subdivision of a FreeSurfer ROI was performed on a template MR image using a *k*-means clustering algorithm based on their intensity similarity (Raamana et al., 2015). Following the *k*-means clustering step, a high-dimensional accurate non-rigid registration method, LDDMM (Beg et al., 2005), was applied to register each ROI of a target MRI to the corresponding ROI of the template. With the ROI-wise registration maps, the patch-wise segmentation of each template ROI was propagated back into the target space. Finally, in the feature extraction and normalization step, the volume of each patch was extracted as a primary feature for disease classification. The w-score, which represents the standardized residual of the chosen features, was computed to remove the effect of covariates such as the field of strength (1.5T or 3T), scanner type, scanning site, age, sex, and the size of the intra-cranial vault (ICV) of each individual (Ma et al., 2018 and Popuri et al., 2020). The normalized features as represented by the w-scores were input into the classifier.

The patch-wise cortical thickness features were extracted in a similar manner to the patch-wise volumetric features. The vertex coordinates in each of the 68 cortical ROIs were subdivided into smaller patches by grouping them with *k*-means clustering based on the pairwise Euclidean distance of their thicknesses in the template space (Raamana et al., 2015). The locally-clustered cortical patches were then propagated back to each of the target space following the backward deformation field that was derived during the LDDMM non-rigid registration step (Beg et al., 2005). The average thickness of the mantle within each patch was computed as features followed by the w-score normalization (Ma et al., 2018 and Popuri et al., 2020) to remove the confounding effect of covariates.

To avoid losing discriminant information during data down-sampling, multiple scale features were extracted in a coarse-to-fine manner. Each ROI was parcellated into three different scales of patch-sizes: 500, 1,000, and 2,000 voxels per patch for the volume features and 500, 1,000, and 2,000 vertex per patch for the thickness features. Those sizes were predefined to retain enough detailed information while restraining the number of primary features with respect to the number of training data to prevent overfitting. The subdivision of ROIs into these three scales resulted in a total number of 1,488, 705, and 343 voxel patches for the gray matter volume feature, and a total number 527, 255, and 131 vertex patches for the cortical thickness feature, respectively. Together with the FreeSurfer ROIs providing volumes and thickness, this gives six feature sets containing 3,409 scalars that represent each brain MR image.

### 2.3. Deep Neural Network for Multi-Scale and Multi-Type Feature Combination

With the patch-wise volume size and surface thickness features extracted from MRI images, a multi-scale and multi-type feature deep neural network (MMDNN) was constructed to learn the latent pattern from both types of features for the classification of NC, AD, and FTD pathology, which achieved state-of-the-art binary classification of NC and AD subjects using both FDG-PET and MRI images in our previous study (Lu et al., 2018a).

**Figure 1:**
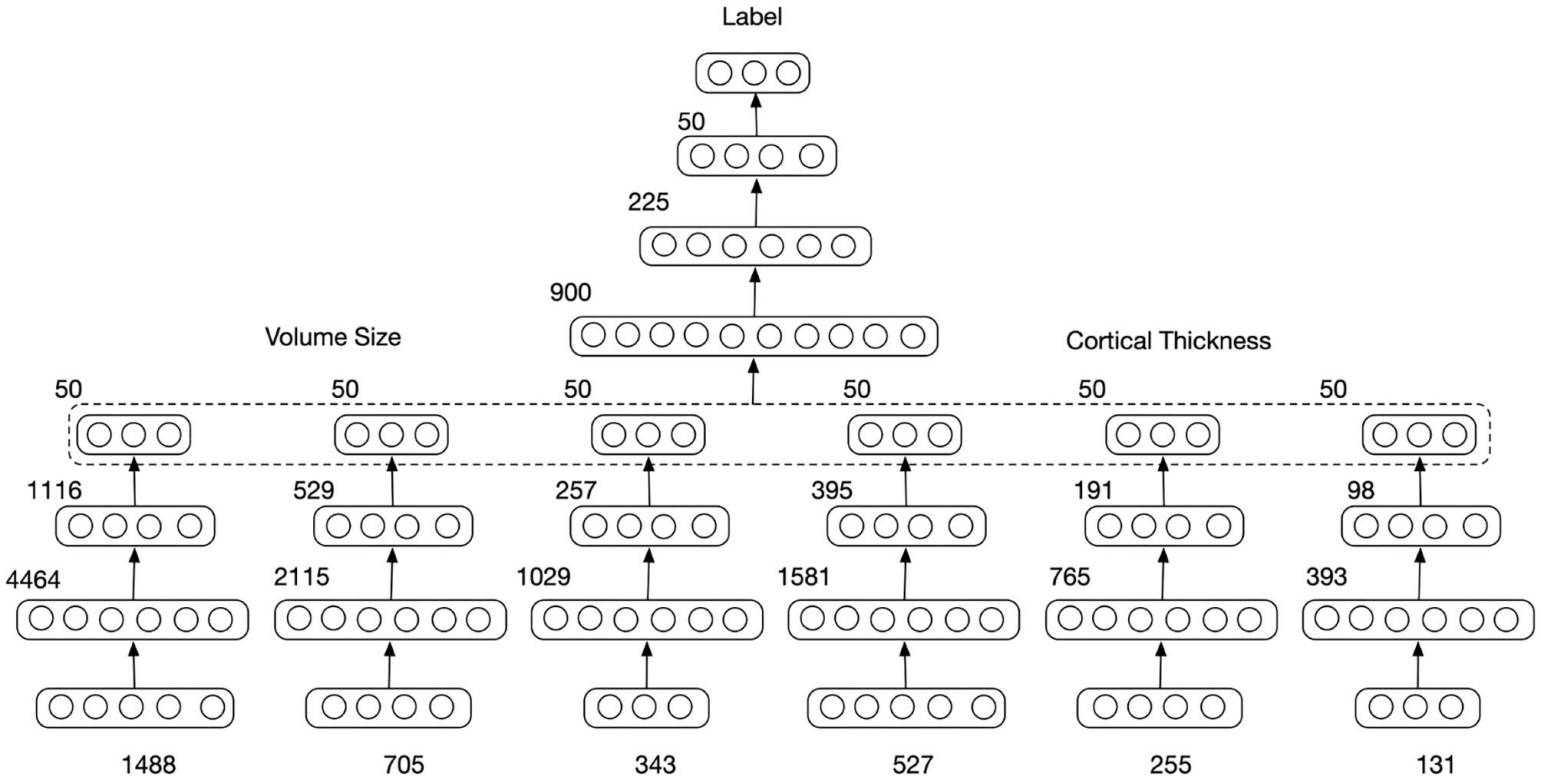
Multi-scale and multi-type feature Deep Neural Network. The input feature dimension (number of patches) extracted from different scales is 1,488, 705, and 343 for volume of gray matter ROI and 527, 255, and 131 for the cortical thickness. The numbers of units in each layer representation are displayed on the top left.

As displayed in Figure 1, the MMDNN consisted of two stages with a total of seven blocks Multilayer Perceptrons (MLPs). The first network stage consisted of 6 MLPs blocks, each corresponds to a single type of features extracted at a single scale. These MLPs were trained independently in the first stage, and their outputs were concatenated as the input feature vector to train the final MLP block in the second stage. The parameters of the whole network were then fine-tuned together. For each image, the output was three probabilities with each corresponding to a subject group, i.e., NC, AD, and FTD, and the class with the highest probability was deemed to be the resulting classification. For each MLP, the number of units for each layer are displayed on its top left in Figure 1. If the dimension of input feature is represented with *N*, the number of units in a single MLP were predefined as 3*N*, $\frac{3}{4}N$, and 50 to increase the chance of exploring a larger range of potential hidden correlations across different patches in the first layer and gradually reduce the number of features in the following layers to avoid too many parameters Lu et al. (2018b,a).

To avoid overfitting, dropout layers (Srivastava et al., 2014) were added after each hidden layer. During the training stage, half of the units were randomly dropped to prevent complex co-adaptations on training data as well as to reduce the amount of computation and improve the training speed. During the validation or testing stage, all the units were retained to feed features to the next layer.

### 2.4. Data Augmentation With Generative Adversarial Network

In deep/machine learning, a common strategy to increase the number of training samples and prevent overfitting is data augmentation. Operations, such as rotation, flip, and zooming, are commonly used for 2D image recognition. However, those operations can hardly be used on a 1D feature vector. GAN (Goodfellow et al., 2014) have emerged to be a powerful tools to synthesize new data and have gained popularity in the generation of realistic natural images, and has also shown great potential to be a powerful data augmentation technique to synthetic image data with more variation and improve the generalizability of the machine learning algorithm (Shi et al., 2018; Lata et al., 2019; Sandfort et al., 2019; Shao et al., 2019). Therefore, we investigated the possibility of applying GAN for 1D structural brain feature augmentation for the improvement of classification performance in this study.

**Figure 2:**
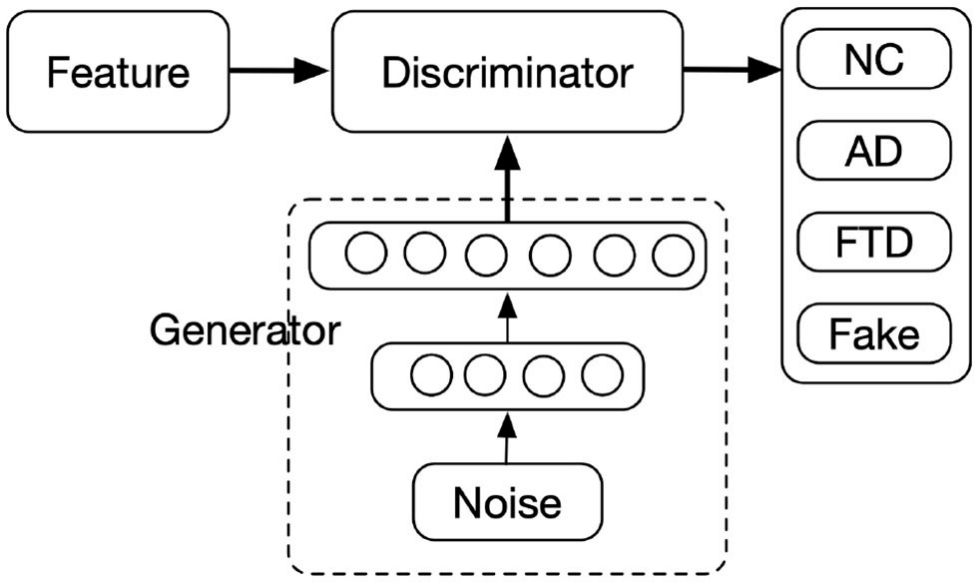
Architecture of generative adversarial network. The numbers of units of Generator layers are 512 and 3,449(1,488+705+343+527+255+131), respectively.

GANs consist of two parts, the Discriminator (D) and the Generator (G), as displayed in Figure 2. In the proposed framework, the MMDNN was used as the discriminator with an additional channel of output for the recognition of data synthesized by the generator, denoted here as “fake,” while the generator aimed to generate feature vectors to “fool” the Discriminator, i.e., classified as NC, AD, or FTD by the discriminator. The input of the generator was a 1D random noise vector. By finding the mapping from the random variables to the data distribution of interest, the generator outputs a feature vector with the same dimension as the real data samples. It was worth mentioning that the fourth channel of output was only used during the optimization of GAN. For each testing sample, only the output probabilities of the first three channels were used to determine which of the three groups a subject belongs to.

To prevent potential problems due to vanishing gradients, the generator consists of two layers, a single hidden layer and an output layer. Both layers are fully connected layer with 512 and 3,449 units, respectively. The dimension of random noise was set to 100 with each element set to follow a normal distribution. The activation function for the first layer was a rectified linear unit (ReLU) to avoid gradients from vanishing, while the one for the second layer was tanh function to squash the synthesized data into the same range of the real data.

### 2.5. Network Optimization

For optimization of the GAN, the loss function was defined:

(1) $$\begin{array}{l} \underset{D}{\min}\underset{G}{\max}V\left(D,G\right)={𝔼}_{x\sim p_{data}\left(x\right)}\left[logD\left(x\right)\right]\\ \;+{𝔼}_{z\sim p_{z}\left(z\right)}\left[log\left(-D\left(G\left(z\right)\right)\right)\right] \end{array}$$

where *x* represents the input data and *p*_*z*_(*z*) is the prior of input noise variables. *log*(−*D*(*G*(*z*))) was used instead of *log*(1 − *D*(*G*(*z*))) to avoid vanishing gradient and mode collapse (Arjovsky and Bottou, 2017). The *E* here stands for weighted cross entropy function, which is defined as:

(2) $$\begin{array}{l} E(logD(x))=-\frac{1}{N}\sum_{i=1}^{N}\sum_{j=1}^{4}\left[𝟙\{y^{i}=j\}W^{j}log(h{\left(x^{i}\right)}_{j}\right] \end{array}$$

where *N* is the number of input samples, *j* represents the class of samples, *W*^*j*^ stands for the weight of class *j* which is computed as the inverse proportion of the subject number for the current class over the entire sample data, *x*^*i*^, *y*^*i*^ are the feature vector and label of sample *i*, and *h* represents the network function.

For the training of GAN, the discriminator and the generator were optimized alternately. During the optimization of the discriminator, the parameters of the generator were held constant, and when the generator was trained, the parameters of the discriminator were fixed. The minimax competition between *G* and *D* could drive both networks toward better performance.

Besides adding dropout layers, another strategy, early stopping, was applied during the training process to reduce the overfitting. During the training of the deep neural network, iterative back propagation could drive the network to co-adapt to the training set. After a certain point, reducing training error could result in increasing the generalization error. Early stopping was therefore useful to provide guidance for the number of optimization iterations before overfitting. Part of the training data was randomly selected as the validation set and were excluded from training. While the remaining data samples were used to train the network, the validation set was used to determine the early stopping time point: the iteration in which the network has the lowest generalization error for the validation set. In this study, optimization of the network was stopped when the generation error of the validation set ceased to decrease for a consecutive 20 epochs.

Furthermore, due to the limited number of available data and variation among different samples, there was still a chance that early stopping with a small validation set could result in biased classification toward the validation set, and the differential performance could be unstable with different splitting of training and validation sets. An ensemble classifier strategy (Lu et al., 2018b) was therefore used to improve the robustness, stability, and generalizability of the classifier. Similar to the 10-fold cross validation, the training set was randomly divided into 10 subsets. In each fold of the training process, one subset was retained for validation while the remaining nine subsets were used for training. With 10 repetitions, each set was used for validation once resulting in 10 different networks. For each test sample, each network would generate three probabilities corresponding to NC, AD, and FTD. The output probabilities of 10 networks were averaged followed with a softmax operation to determine the final classification result.

The proposed deep neural network was built with Tensorflow (Abadi et al., 2015), an open source deep learning toolbox provided by Google. For the optimization of network in all experiments, Adaptive Moment Estimation (Adam) was used as the optimizer, batch size was set as 100 and the learning rate was fixed as 5 × 10^−5^.

### 2.6. Performance Evaluation

To validate the discriminant ability of the proposed framework on NC, AD, and FTD pathology, 10-fold cross validation was performed on the 1,954 T1 MRI images. Because a single subject could have multiple scans at different visits, a split based on images could result in having scans from the same subject used for both training and testing. We therefore performed the split based on subject to ensure complete separation between training and test samples. As mentioned in the section 2.5, the training set was further sub-dived into 10 subsets for each cross validation experiment and 10 networks optimized with different training and validation set were used to “vote” for the classification result of testing samples. Such an experimental design ensures that the data samples in the training, validation, and testing set were mutually exclusive on a subject level. The performance of classification was measured via accuracy and the sensitivity of correctly identifying different groups, such as *N*(*TrueNC*)/*N*(*NC*) for NC group, where *N*(·) denotes the number of data samples belonging to this group.

Other than the proposed deep-learning-based method, a standard classifier, support vector machines (SVM) were also trained for comparison. One vs. rest strategy was applied for this multiclass classification task. Principal component analysis (PCA) was used for the reduction of feature dimension and the eigenvectors accounting for 95% of the total data variance were retained. Radial basis function (RBF) kernel was used for SVM given its superior performance in classification tasks. The features extracted at different scales were concatenated as the input for PCA+SVM classifier. In addition, to validate the effect of patch-wise parcellation, we also trained the MLPs on FreeSurfer ROI-wise features, i.e., the surface thickness and volume size of each ROI based on the Freesurfer segmentation.

## 3. Results

### 3.1. W-Score Feature Extraction

**Figure 3:**
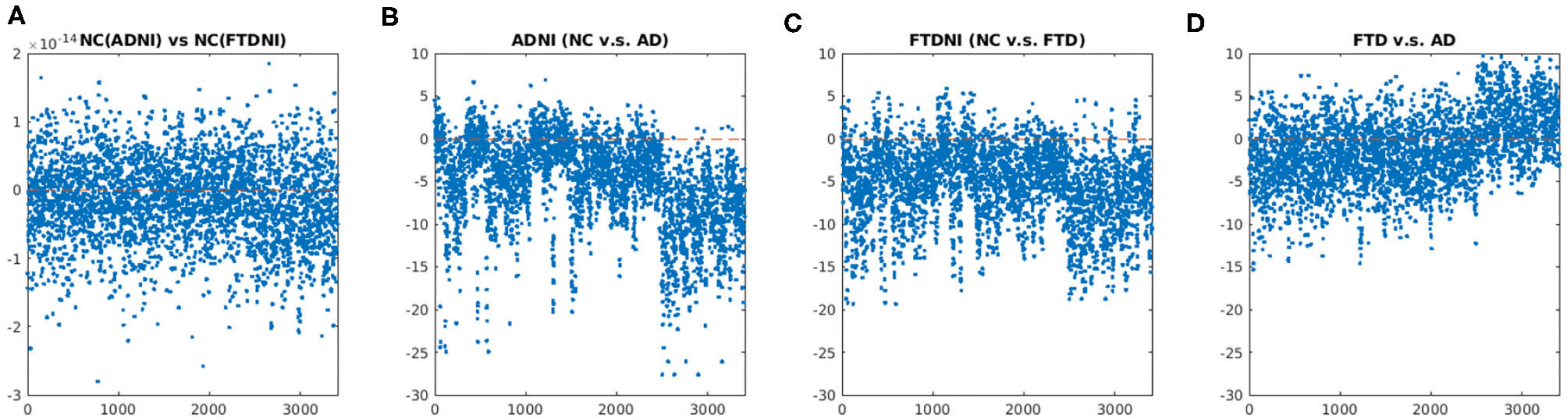
Comparison about the distribution of the concatenated multi-level multi-type W-score feature set among different disease groups: **(A)** NC(ADNI) vs. NC(FTDNI); **(B)** NC vs. AD in ADNI database; **(C)** NC vs. FTD in FTDNI database; and **(D)** FTD vs. AD. **(A)** No statistical difference was shown when comparing the W-scores of the Healthy Control subjects between the ADNI and FTDNI, confirming no database-specific biases remained in the input w-score feature of the normative group. **(B)** Similar level of significant differences were shown when comparing the NC and AD subjects in the ADNI database, or **(C)** When comparing the NC and the FTD subjects in the FTDBI database, indicating similarity between the AD and FTD group. **(D)** When comparing the FTD and AD group alone, significant differences were observed in both the volume-based and thickness-based features, indicating discrepancy between these two types of Dementia subtypes which can be utilized to achieve potential differential diagnosis. Unpaired *t*-test were performed for each pair of the comparison, with multiple comparison corrected by setting false discovery rate (FDR) = 0.05.

Figure 3 showed the comparison of the distributions for the entire concatenated multi-level multi-type W-score feature set between different subgroups. First, no statistical difference were shown when comparing the W-scores of the healthy control subjects between the ADNI and FTDNI for either the volume-based or thickness-based features (Figure 3A), confirming no database-specific biases remained in the input w-score feature of the normative group. Similar level of significant differences were shown when comparing the NC and AD subjects in the ADNI database (Figure 3B), or when comparing the NC and the FTD subjects in the FTDBI database (Figure 3C), indicating similarity between the AD and FTD group. Finally, when comparing the FTD and AD group alone, significant differences were observed in both the volume-based and thickness-based features, indicating discrepancy between these two types of Dementia subtypes, which can be utilized to achieve potential differential diagnosis.

### 3.2. Cross Validation Experiment Results

The results of 10-fold cross validation experiment are shown in Table 2. When comparing the mean accuracy across 10-folds, the accuracy of PCA+SVM with both type of multi-scale features was only slightly higher (0.02%) than the multi-scale deep neural network (MDNN) with surface thickness feature. The accuracy of MDNN using volume size feature was higher than the one using surface thickness feature by 2.93%. The combination of both type of multi-scale features showed superior performance comparing with MDNN using a single type of feature, and it was further improved by 1.42% with the data augmentation using the proposed GAN technique.

**Table 2:** Comparison of classification performance over different experiments with multi-type features.

	**Accuracy**	**NC sen**.	**AD sen**.	**FTD sen**.
PCA+SVM (Multitype)	83.06	93.90	71.74	68.23
MDNN+Thickness	83.04	89.07	76.77	74.79
MDNN+Volume	85.97	91.05	83.88	74.20
MMDNN (Multitype)	86.81	93.76	81.94	73.59
GAN (Multitype)	88.28	93.40	84.66	77.82

*The second column is the overall classification accuracy, while the third to fifth columns represent the sensitivity of NC, AD, and FTD, respectively. The second row represents the result with PCA+SVM using multi-type multi-scale features. The third and fourth rows are the classification performance of MDNN with multi-scale surface thickness or multi-scale volume size. Experiment results with both types of features using MMDNN are shown in the fifth row and the last row represents the result of multi-type multi-scale features along with data augmentation using GAN*.

**Figure 4:**
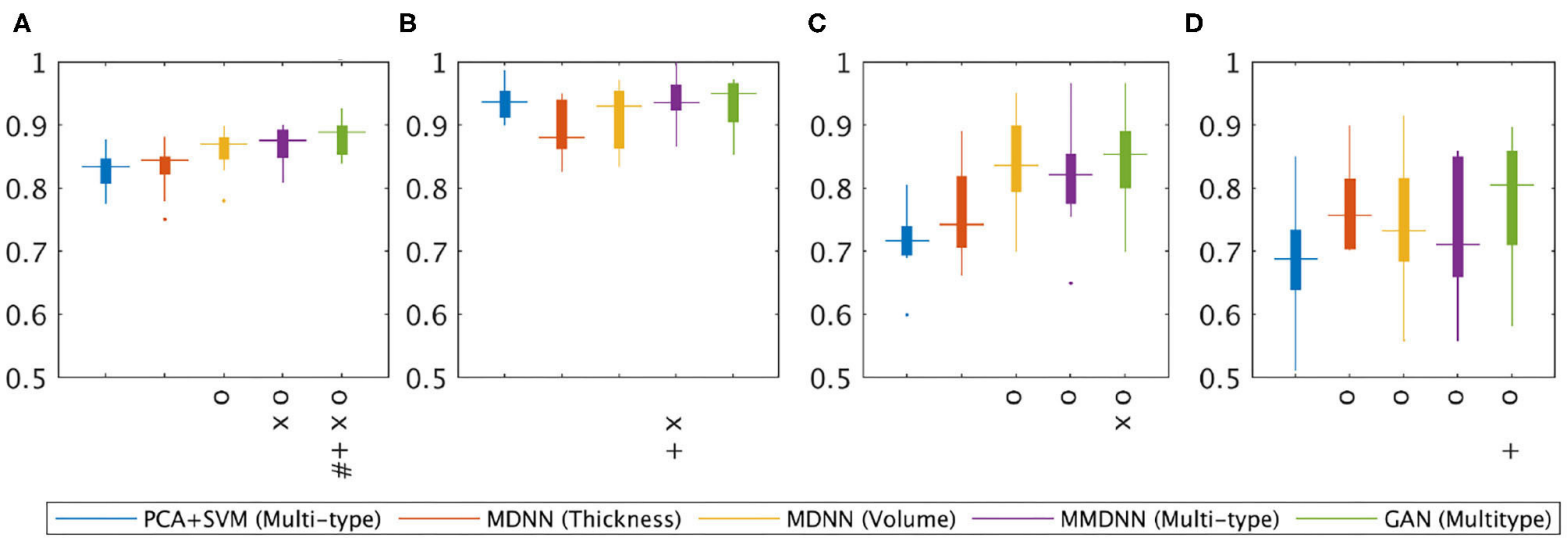
Statistical analysis of the classification performance among different experiments. One-tailed pairwise *t*-tests were conducted to access the performance improvements. Multiple comparisons were corrected with False discovery rate FDR = 0.05. **O**: Significant improvement over PCV+SVM(Multi-type); **X**: significant improvement over MDNN (thickness); +: significant improvement over MDNN (volume); #: significant improvement over MMDNN (Multitype). **(A)** Overall accuracy, **(B)** NC sensitivity, **(C)** AD sensitivity, **(D)** FTD sensitivity.

Figure 4 showed the corresponding statistical comparison results among different experimental setup for the overall accuracy as well as the sensitivity for each class group. When compared to the baseline method, PCA+SVM (multi-type), both the proposed MMDNN method with or without GAN showed significant improvement (indicated as **O**) for the overall accuracy (Figure 4A), as well as sensitivity for AD (C) and FTD (D). Training with multi-type feature showed improvement over the training with only single feature (for either thickness, indicated as **X**, or volume, indicated as +) in terms of overall accuracy (Figure 4A). Finally, data augmentation using GAN further improve the overall accuracy (Figure 4A) as well as sensitivity for the NC group (Figure 4B) and the FTD group (Figure 4D) (indicated as +).

For detailed classification result, the confusion matrices of experiments using the proposed multi-scale networks are displayed in Table 3. The presented four experiments show a similar pattern despite the differences in their accuracy and sensitivity. The networks had a good performance for the task of distinguishing between AD and FTD pathology. The discrimination between NC and FTD showed the least accurate performance, leaving room for potential future improvement.

**Table 3:**
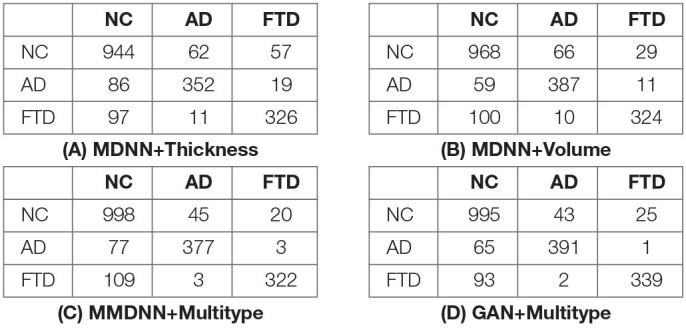
Confusion matrix of GAN.

*The class names of the first column in each table represent the ground truth, and the names in the first row denote the classification result*.

### 3.3. Discrimination With Cortical Thickness Feature

The experiment performance with only cortical thickness feature was displayed in Table 4. MLP with only ROI-wise cortical thickness feature showed the least accuracy (76.48%), while better result was achieved with PCA+SVM using features extracted at all scales. As expected, the classification performance was sensitive to patch size change and a generalized reduction with increasing patch size was found on the overall accuracy. The combination of multi-scale features with MDNN yielded superior classification performance.

**Table 4:** Comparison of classification performance over different experiments with cortical thickness feature.

	**Accuracy**	**NC sen**.	**AD sen**.	**FTD sen**.
PCA+SVM	81.12	91.04	73.78	63.43
ROI MLP	76.48	82.74	71.80	66.55
500 MLP	82.80	87.95	77.02	76.05
1000 MLP	81.22	86.92	72.13	72.48
2000 MLP	79.51	84.49	75.84	71.20
MDNN	83.04	89.07	76.77	74.79

*The second column is the overall classification accuracy, while the third to fifth columns represent the sensitivity of NC, AD, and FTD, respectively. The second row represents the result using PCA+SVM with multi-scale surface thickness features, while the third row shows the classification performance of a single MLP with ROI-wise features. The fourth to sixth rows are the result of a single MLP with features extracted at different scales, i.e., 500, 1,000, and 2,000 voxels per patch. The last row represents the MDNN result with multi-scale surface thickness features*.

### 3.4. Discrimination With Volume Size Feature

The experiment performance with volume size feature was displayed in Table 5. Similarly, as the experiments with cortical thickness feature, MLP with only ROI-wise feature had the worst performance (79.78%), and PCA+SVM using features extracted at all scales showed better accuracy (82.28%). Unlike the experiments with cortical thickness feature, MLP with a single scale of feature showed better performance comparing with PCA+SVM using features extracted at all scales. The combination of multi-scale features with MDNN also had the highest accuracy, while no generalized reduction of accuracy was found with increasing of patch size.

**Table 5:** Comparison of classification performance over different experiments with ROI volume Feature.

	**Accuracy**	**NC sen**.	**AD sen**.	**FTD sen**.
PCA+SVM	82.28	85.94	85.44	67.14
ROI MLP	79.78	83.44	79.79	69.67
500 MLP	85.78	91.60	82.83	73.31
1000 MLP	85.41	90.03	84.91	73.07
2000 MLP	85.45	90.34	82.26	75.06
MDNN	85.97	91.05	83.88	74.20

*The second column is the overall classification accuracy, while the third to fifth columns represent the sensitivity of NC, AD, and FTD, respectively. The second row represents the result using PCA+SVM with multi-scale volume size features, while the third row shows the classification performance of a single MLP with ROI-wise features. The fourth to sixth rows are the result of a single MLP with features extracted at different scales, i.e., 500, 1,000, and 2000 voxels per patch. The last row represents the MDNN result with multi-scale volume size features*.

### 3.5. Ensemble Classifier

**Figure 5:**
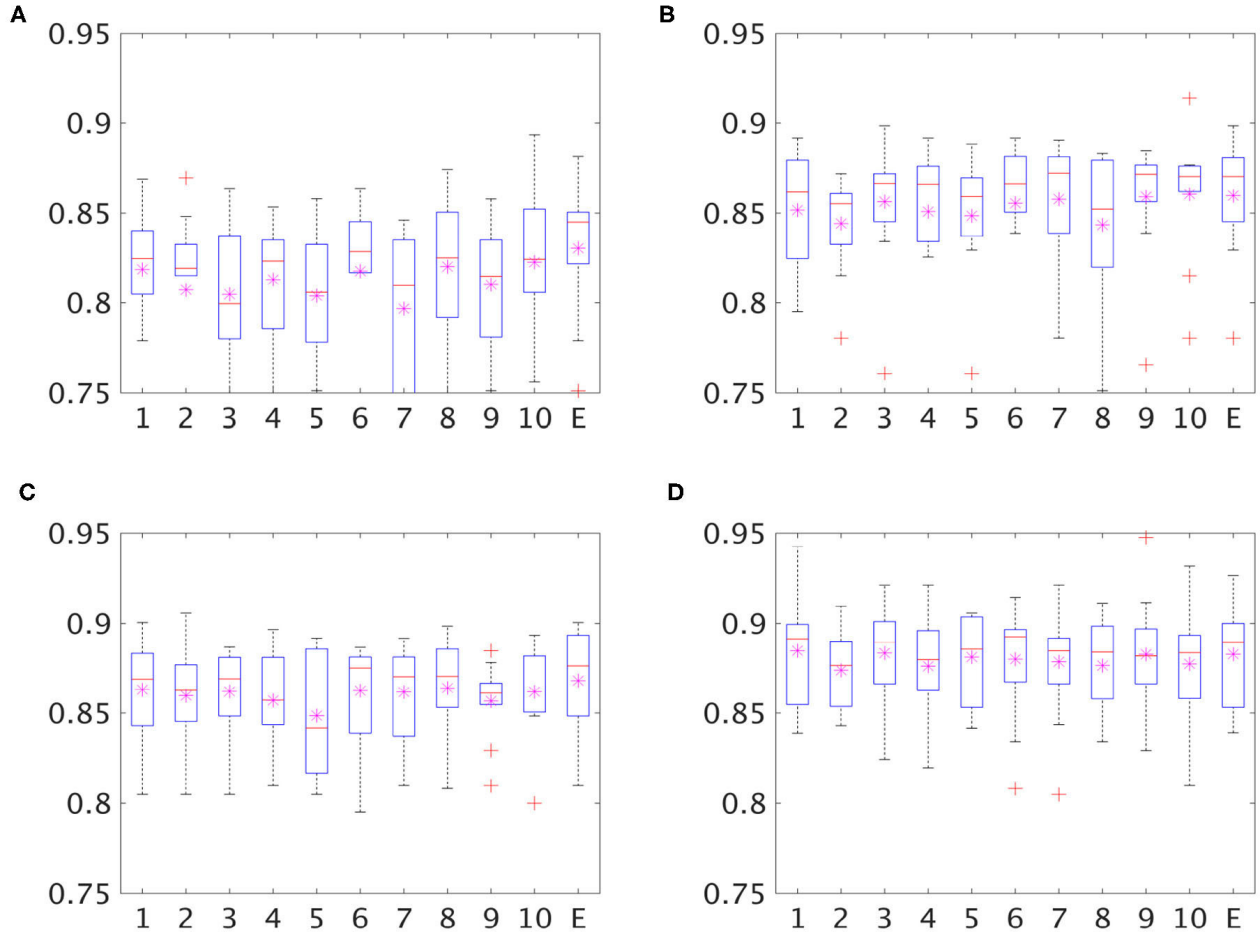
Boxplot for classification accuracy of single classifiers (classifier 1–10 on *x* axis) and an ensemble of classifiers (E on the *x* axis). The stars in each box are the mean of accuracy and the red lines represent the median accuracy. **(A)** MDNN+Thickness, **(B)** MDNN+Volume, **(C)** MMDNN+Multitype, **(D)** GAN+Multitype.

As described in section 2.5, the classification results presented in this study came through the “collective vote” of an ensemble of classifiers instead of a single network. The classification performance with or without ensemble classifiers of four different experiments, including MDNN with cortical thickness, MDDN with volume size, MMDNN with multi-type of features and GAN with multi-type of features, are shown in Figure 5. The *y* axis represents the mean classification accuracy from the 10-fold cross validation experiment, while the *x* axis stands for different classifiers. On the *x* axis, the number “1” to “10” represents the network trained with different split of training and validation set, while “ensemble” denotes the combined result of these 10 networks.

## 4. Discussion

In this study, we proposed a novel deep-learning-based framework for the differential diagnosis of NC, AD, and FTD. Cross validation experiment indicate that the proposed network could learn the latent patterns representing the different dementias using multi-type and multi-scale features, which in combination with GAN-based data augmentation, achieved a high accuracy of 88.28%. Based on the confusion matrix displayed in Table 3, there were only three cases of misdiagnoses between AD and FTD out of 891 samples, suggesting the excellent performance of the proposed framework to distinguish these two dementias.

### 4.1. Differential Diagnosis Using MRI Biomarker

Brain MRI is an imaging modality widely used for detecting various types of dementia, as the image contrast between different tissue can reveal pathology-induced brain morphology changes. Due to variations in pathogenesis and phenotypes, dementia can further be categorized into different sub-types, such as FTD, AD, mild cognitive impairment, vascular dementia, and dementia with Lewy bodies. Differentiating among different dementia subtypes is crucial for to provide appropriate healthcare and potential treatment, but is challenging due to overlapping phenotyping and morphological heterogeneity with each subtype (Bruun et al., 2019), and accurate differential diagnosis requires both appropriate feature extraction technique combined with powerful classification model. Some recent studies attempted to differentiate dementia subtypes using different machine learning techniques, such as hierarchical classification (Kim et al., 2019), statistical learning with feature selection based on least absolute shrinkage and selection operator (LASSO), and support vector machine (SVM) (Zheng et al., 2019), but are limited from either the constrained feature set (e.g., structural-volume feature) or relatively small validate with data for testing the robustness and generalizability of the classifiers. In our study, we proposed a framework to achieve accurate differential diagnosis by first building a multi-scale multi-type feature, followed with a deep neural network with the help of generative adversarial data augmentation technique, which was validated on a large sample (1,954 images), demonstrating a consistent overall high accuracy.

### 4.2. Multi-Scale Classification

Based on the results presented in Table 4, the accuracy of MLP decreased from 82.80% to 79.51% with patch size increasing from 500 voxels to 2,000 voxels, suggesting that cortical thickness feature is sensitive to the change of size of the ROI patch sizes, while less variation of accuracy was found with ROI volume feature (from 85.78% to 85.41%) as shown in Table 5. Contradicting our observations on using cortical thickness feature, the accuracy of volume size feature showed a slight improvement when the patch size increased from 1,000 to 2,000 voxels, suggesting that the volume change caused by brain atrophy may affect a large brain region in a similar fashion. However, the combination of multi-scale features always resulted in a better classification performance, indicating that the proposed MDNN is capable of learning the hidden pattern across the small to large patch sizes regardless the feature type. The optimal scale with the best performance would be a potential tunable hyperparameter in an optimization framework.

### 4.3. Volume Size, Surface Thickness, and Other Morphological Features

Two types of features, ROI volume and cortical thickness, were used for differential diagnosis in this study. Cross validation experiments showed that volume size has better discriminant ability compared with surface thickness regardless of the scale of feature and the type of classifier, as presented in Tables 4, 5. In addition, the results in Table 2 show that with the same classifier, the combination of these two features yields superior classification performance comparing with single type of feature, regardless of whether they are concatenated as a single input feature vector for SVM or using a MLP to learn the latent representation of each scale of feature first.

In this study, we have explored the extraction volume-based and cortical-thickness-based features as an effort to improve the power of differential diagnosis. Other additional image-based morphological features could potentially also provide complementary information regarding brain pathology. Specifically, cortical folding has showed different aging-related patterns between healthy and diseased brain (Wang et al., 2016), including dementia such as AD (Cash et al., 2012). The combination of cortical folding with other shape-based descriptors such as local cortical thickness could potentially yield better characterization the cortical morphological changes that is induced by AD and other types of dementia (Awate et al., 2017). Therefore, the proposed framework could potentially be further extended to integrate other brain morphological descriptors, such as the cortical folding, into the multi-type input feature space to achieve better classification and differential diagnosis power.

In the current study, the proposed network was trained using structural-MRI-based patch-wise volume size and surface thickness features created with a combination of from FreeSurfer segmentation and k-mean clustering to balance the number of parameters trainable and the level of original image-based patterns that are preserved. A potential future direction is to learn the features directly from the raw structural image while maintaining a trainable number of network parameters, which still remains a challenge. This study with patch-wise FreeSurfer-segmentation-based features sets a baseline benchmark for future studies of deep-learning-based differential diagnosis studies with novel network-leaned image-based features for comparison.

### 4.4. Data Augmentation With GAN

As displayed in Table 2, the classification accuracy was further improved by 1.42% when using GAN for data augmentation. The sensitivity for detecting AD and FTD pathology was increased by a large margin with a slight decrease for detecting NC samples. Instead of *log*(1 − *D*(*G*(*z*))), we used *log*(−*D*(*G*(*z*))) in loss function to avoid vanishing gradient and mode collapse (Arjovsky and Bottou, 2017). Therefore, we did not specify what kind of data samples the generator should synthesize. We consider it as a “success” for the generator as long as the generated feature vector was classified as one of the three categories, i.e., NC, AD, and FTD, by the Discriminator. It would be interesting to train one or three Generators to synthesize data samples corresponding to specific groups, although this is beyond the scope of this study as our primary goal was to increase the differentiating accuracy.

For the generator, we only have a single hidden layer because of the low dimension of our data and potential gradient vanishing problem. Instance normalization or other kinds of normalization (Almahairi et al., 2018) was not performed because they caused mode collapse of the generator and resulted in synthetic data all close to 0. Contrasting with many other studies using GAN (Arjovsky et al., 2017), we found root mean square propagation (RMSprop) optimizer resulted in an 87.39% accuracy, which was lower than with Adam optimizer.

### 4.5. Ensemble Classifier and Cross-Validation

As shown in Figure 5, there can be as much as 3% difference in the classification accuracy (the seventh and the tenth bar of the top left image) across the individual classifiers trained with a different subdivision of the training and validation set, suggesting an unstable performance of each single classifier. In all four experiments, the ensemble classifier had the highest or close to highest accuracy, suggesting that the ensemble strategy improves the robustness and generalizability of the classifier.

It was worth mentioning that with the GAN, the variation of classification accuracy with individual classifiers decreased to 0.49% (from 87.98 to 88.47%) while the accuracy of ensemble classifier was 88.28%, suggesting that, with using GAN for data augmentation, the complex co-adaptations to training or validation set were reduced. The ensemble classifier strategy, although still effective, could therefore be optional with the application of GAN in light of limitations of available computational resources.

On top of the combination of GAN-based data augmentation and cross-validation-based ensemble classifier, an additional nested 10-fold cross validation was implemented to ensure the proposed method is properly validated. Nevertheless, it would be ideal to validate the proposed multi-class classifier on an entire independent and well-homogenized dataset to best evaluate its generalizability toward unseen dataset (Popuri et al., 2020; Yee et al., 2020).

## 5. Conclusion

In this study, a novel framework for accurate differential diagnosis among NC, AD, and FTD pathology has been proposed leveraging the multi-type and multi-scale feature fusion, ensemble classifier, and GAN strategy. The proposed framework achieved a high accuracy of 88.28%. The cross-validation experiments conducted on 1,954 MRI images demonstrate three salient observations. Firstly, the proposed network was able to learn the latent representation pattern across the different types of features (volumes and cortical thickness) extracted at coarse-to-fine scales. Secondly, using a Generative Adversarial Network for data augmentation could prevent overfitting and improve classification performance. Thirdly, the ensemble classifier strategy could result in a more robust and stable classifier, which has statistically better performance than an individual classifier. The promising high-accuracy results using the proposed framework, and the ability of deep networks to generalize to multiple classes, indicate that this approach can be potentially extended for the multiclass differential classification of brain images in other neurodegenerative dementias as well.

## Data Availability Statement

The datasets generated for this study are available on request to the corresponding author.

## Author Contributions

DM conducted the experiment, performed the data processing and analysis, and wrote the manuscript. LW and MB designed and supervise the experiments, guided, and revised the manuscript. KP performed the data processing and manuscript writing. DL conducted the experiment, designed the framework, performed the analysis, and wrote the manuscript. All authors contributed to the article and approved the submitted version.

## Conflict of Interest

The authors declare that the research was conducted in the absence of any commercial or financial relationships that could be construed as a potential conflict of interest.
